# Ten years of screen time among medical students in Dresden, Germany: unveiling the trends

**DOI:** 10.1186/s12909-026-09024-x

**Published:** 2026-03-30

**Authors:** Lukas Liebig, Willy Gräfe, Nicholas Lynch, Mandy Gottschall, Béla Birkás, Erika Balogh, Nora Faubl, Erika Zelko, Henna Riemenschneider

**Affiliations:** 1 Department of General Practice, Faculty of Medicine Carl Gustav Carus, TUD Dresden University of Technology, Fetscherstraße 74, Dresden, Saxony 01307 Germany; 2 Student, TUD Dresden University of Technology, Dresden, Saxony Germany; 3 Department of Behavioural Sciences, University of Pécs Medical School, Pécs, Hungary; 4 Department of Public Health Medicine, University of Pécs Medical School, Pécs, Hungary; 5 Institute of General Practice, Johannes Kepler University Linz, Linz, Austria

**Keywords:** Screen time, Medical students, Media use, Longitudinal analysis, Generalized linear model, Screen use, Medical school, Germany, Trends, Risk factors

## Abstract

**Introduction:**

Screen time has become an integral part of academic and private life, especially for medical students, whose daily routines increasingly depend on digital devices. Prolonged screen time is associated with negative health outcomes. There is no long-term data on trends in screen time among medical students in Germany.

**Methods:**

Data from *n* = 2656 medical students at TU Dresden were collected biennially (2014–2024) via the Medical Student Health Survey. Screen time for study/work and leisure was self-reported. Data were analyzed descriptively and via Generalized linear models to analyze screen time trends and associated factors.

**Results:**

Total daily screen time increased from M = 5.1 h (2014) to M = 7.7 h (2024) (+ 51.0%). Study/work-related screen time rose from M = 3.31 h to M = 5.23 h per day (+ 58.0%), and leisure screen time increased from M = 1.79 h to M = 2.46 h per day (+ 37.4%). Significant predictors of screen time included survey year, study phase, gender, age and living situation.

**Discussion:**

Medical students’ screen time has increased significantly over the past decade. Increased screen time is associated with adverse physical and mental health outcomes, including eye strain, impaired sleep, reduced cognitive performance, and diminished emotional well-being. Evidence-based interventions promoting digital health literacy and healthg-conscious screen use are needed to protect student health.

**Supplementary Information:**

The online version contains supplementary material available at 10.1186/s12909-026-09024-x.

## Introduction

Screens are omnipresent in daily life, both during the day and at night, and are used throughout school, university, work, and leisure time. The time spent in front of screens—such as computers, tablets, smartphones, televisions, or gaming consoles—is referred to as screen time [15]. Screen time is often performed in a sedentary manner, which has been linked to cardiovascular diseases [20], depression [23, 51] and a higher all-cause mortality [39, 52]. Moreover, excessive screen time can lead to multiple consequences, such as impaired sleep [17, 32] and reduced cognitive function such as attention span or memory [31, 37]. Despite these concerns, there is currently no official guideline in Europe regarding screen time for individuals over the age of 18.

Digitalization has significantly increased daily screen time over recent decades [8, 45]. According to the literature, this trend was further accelerated by the COVID-19 pandemic [43]. Medical students were impacted by the COVID-19 pandemic as well. The abrupt transition to online teaching and examinations was particularly challenging given the practical, laboratory, and clinical components of their training [11, 27]. Today, university education is nearly inseparable from screen use, particularly in lecture- and theory-dominated disciplines. This also applies to medical school in Germany, where medical students spend an average of M = 7 h per day in front of a screen [24]. Medical students are a high-risk population for unhealthy behaviors due to their stressful, competitive academic environment. Harmful health behaviour which is prevalent among medical students ranges from substance abuse [4, 47] over bad dietary habits [41, 42] to prolonged screen time [24].

Beyond maintaining their personal health, the healthcare system relies on medical students to complete their studies successfully and in good health. First, well-educated and healthy medical professionals form the backbone of a functioning and resilient healthcare system. Moreover, physicians who adopt health-conscious behaviors are more likely to counsel their patients on such matters and serve as more convincing role models [7, 30].

Prior work indicates that increased screen time among medical students in Germany is associated with worse sleep behaviour [24] or harmful smartphone usage [25].

Thus, the goal of the following research is to unveil the trends in screen time among medical students in the last 10 years. The results will contribute to a better understanding of an increasingly relevant behavioural risk factor, that negatively interacts with psychological and physiological health. These insights are expected to inform the development of future educational and preventive structures which foster the well-being of medical students.

## Methods

Medical students of the Technical University of Dresden (TUD) were surveyed biennially from 2014 to 2024 as part of the multicentric cross sectional study “Medical Student Health Survey” (MSHS). The survey was conducted by the Department of General Practice at the Faculty of Medicine and University Hospital Carl Gustav Carus, Dresden and the Department of Public Health Medicine and the Department of Behavioural Sciences at the Faculty of Medicine of the University of Pécs, Hungary. The primary study sites were Dresden and Pécs. Depending on the survey year, additional sites included Halle, Munich, Budapest, Linz, Graz and Ljubljana. Correspondingly, cooperation with various university departments also varied by survey year. Due to comparison reasons, only data from medical students from the TUD are included in the following analysis.

The students were invited to participate voluntarily. From 2020 onward, participation was pseudonymous. The questionnaire was conducted as paper/pen survey from 2014 to 2018 and administered as an online survey via LimeSurvey from 2020 onward. The survey period was the summer semester of the respective year. The questionnaire consisted of standardised and self-developed items which covered the sociodemographic data, the health status and the health behaviour of the participants. The survey was developed further every survey period and continually pretested to ensure validity and reliability.

Participants were recruited by advertisements, at social media, e-mail-invitations and active recruitments in seminars and lectures. The study was registered by the ethics committee of the TUD (EK15012014). 2014 and 2016 only the semester 2.,6. and 10. were surveyed. In 2018 only the 10. semester and up from 2020 every semester were surveyed. Therefore, the response rate for 2014 to 2018 was calculated by dividing the number of participating medical students by the number of matriculated medical students in the surveyed semesters (2nd, 6th, and 10th). The response rate for 2020 to 2024 was calculated by dividing the number of participating medical students by the total number of matriculated medical students. During the COVID period in 2020 Data collection was carried out between January and July 2020, with a pause from March to May due to the COVID-19 pandemic and related preventive measures.

Screen time was assessed by the same item in every survey period, separately for study/work and for leisure reasons: “O*n average*,* how much time do you spend daily on the computer/tablet PC/mobile phone: …for study/work? …for leisure (e.g. browsing/social media/games/videos/ebook)?” Answer… in hours”. The specified screen time for study/work and* leisure was added up to calculate the daily spent total screen time (TST). No psychometric assessments were performed for the items, but content validity was verified in a pretest with medical students, clinicians, and scientific staff using the think-aloud method.

As various factors interact with screen use, those variables were included which (a) were proven to be associated with screen use by previous conducted research (b) were continuously collected from 2014 to 2024. As a consequence, data about age, gender (f/m), living situation (alone vs. not living alone) and study phase (preclinical vs. clinical) were included in the analysis.

### Statistical analysis

Only students who provided complete and plausible (≤ 18 h total screen time) information on daily screen time were included in the analysis (the cut-off value of 18 h was chosen because it is unrealistic for a person to sleep less than 6 h and spending full wake time in front of a screen on a regular basis). Statistical analysis was performed with IBM SPSS v30 and JASP 0.19.3.

Screen times for study/work, leisure, and total use were analyzed descriptively (mean, standard deviation, median) for each survey year. Percentage changes compared to the previous year were also reported. Normality was tested through Shapiro-Wilk test, skewness and kurtosis values divided by their respective SDs, and visual analysis of Q-Q plots. Since screen time values were treated as continuous variables, generalized linear models (GLMs) with both Gamma and Gaussian distributions were fitted to assess model fit. For the Gamma family, inverse and log link functions were computed.

Since GLMs modelling Gamma families require only positive values greater than zero [10], 0.01 was added as a negligible constant to all values, to ensure leaving substantive estimates unaffected. The number of students with a value of zero is *n* = 9 (0.3%) for study/work screen time, *n* = 69 (2.6%) for leisure screen time, and *n* = 1 (0.04%) for total screen time. Based on these very small numbers, the gamma GLM seems appropriate. Model fit was assessed through comparison of deviance and Pearson Chi-Square values as well as the Akaike Information Criterion (AIC) and the Bayesian Information Criterion (BIC), with lower values for AIC and BIC indicating better goodness-of-fit and adequate parsimony [1]. Additionally, diagnostics for multicollinearity (variance inflation factor (VIF), tolerance, correlations) were computed. Reported estimates were derived from the coefficients and converted to percentages.

## Results

### Study population

Between 2014 and 2024, a total of *n* = 2965 medical students from TUD participated in the Medical Student Health Survey. Complete and plausible data (TST ≤ 18 h) were available for *n* = 2656 participants, who were consequently included in the analyses. Response rates varied between 25% and 87% across the survey years (full data including semester distributions available in Supplement 1). Depending on the survey year, the average age of participants ranged from M = 23.5 to M = 26.0 years, with the proportion of female students varying between 65.0% and 73.1%. An overview of the sample characteristics for each survey year is presented in Table 1.

**Table 1:** Sample description of the survey years 2014-2024

	2014(n=585)	2016(n=478)	2018(n=158)	2020(n=416)	2022(n=655)	2024(n=364)
Age, M (SD)	23.47 (3.45)	23.72 (3.62)	26.01 (3.04)	24.39 (3.92)	24.08 (3.76)	24.06 (3.70)
Gender, n (%)						
Male	199 (34.0)	159 (33.3)	52 (32.9)	115 (27.6)	178 (27.2)	96 (26.4)
Female	380 (65.0)	315 (65.9)	103 (65.2)	292 (70.2)	467 (71.3)	266 (73.1)
Missing, diverse	6 (1.0)	4 (0.8)	3 (1.9)	9 (2.2)	10 (1.5)	2 (0.5)
Study period, n (%)						
Preclinical	217 (37.1)	193 (40.4)	-	142 (34.1)	234 (35.7)	141 (38.7)
Clinical	366 (62.6)	285 (59.6)	158 (100)	234 (56.3)	417 (63.7)	212 (58.2)
Missing	2 (0.3)	-	-	40 (9.6)	4 (0.6)	11 (3.0)
Living situation, n (%)						
Alone	195 (33.3)	143 (29.9)	54 (34.2)	116 (27.9)	224 (34.2)	133 (36.5)
With others	383 (65.5)	335 (70.1)	102 (64.6)	298 (71.6)	426 (65.0)	224 (61.5)
Missing	7 (10.3)	-	2 (1.3)	2 (0.5)	5 (0.8)	7 (1.9)

### Trends in screen time: descriptive findings from 2014 to 2024

**Fig. 1:**
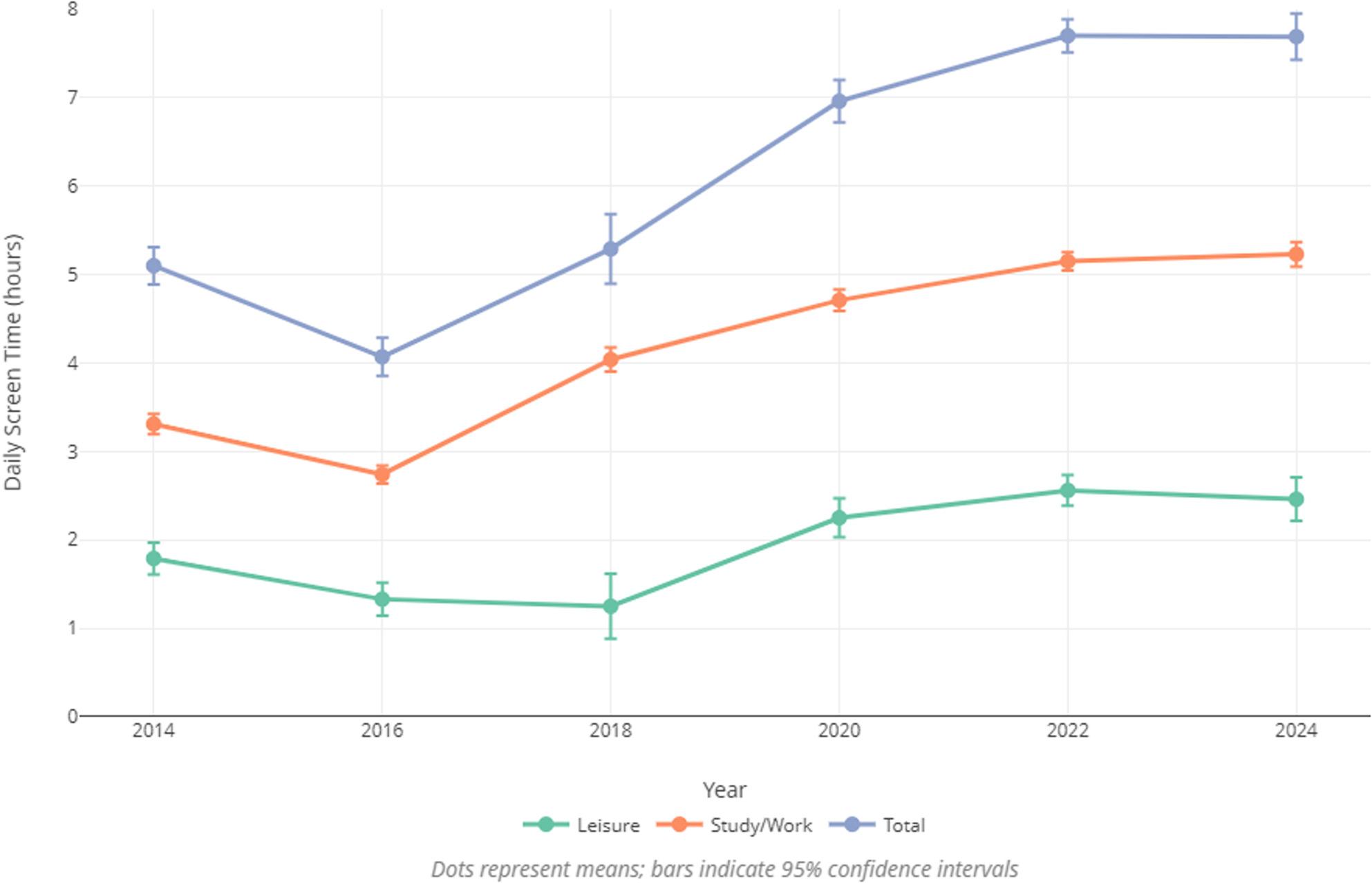
Course of screen time from 2014 to 2024

The average screen time for study and work was M = 3.31 h (SD = 2.23) per day in 2014. In 2016, this value decreased to M = 2.74 h (SD = 2.10), and subsequently increased continuously over the following years, reaching M = 5.23 h (SD = 2.39) per day by 2024 (Fig. 1).

The average screen time for leisure purposes was M = 1.79 h (SD = 1.40) in 2014, decreased to M = 1.33 h (SD = 1.13) in 2016, and further to M = 1.25 h (SD = 0.87) in 2018, before rising to M = 2.46 h (SD = 1.35) by 2024 (Fig. 1).

Average total screen time decreased from 2014 to 2016 (M = 5.10 h (SD = 2.61) vs. M = 4.07 h (SD = 2.40)), and then increased to M = 7.69 h (SD = 2.56) by 2024 (Fig. 1^1^). The respective screen times for each survey year, as well as the percentage change compared to the previous year, are presented in Table 2.

**Table 2:** Screen times (in hours) of the study cohorts 2014-2024

	2014(n=585)	2016(n=478)	2018(n=158)	2020(n=416)	2022(n=655)	2024(n=364)
Screen time – study/work						
Median (Min – Max)	3 (0 – 15)	2 (0.07 – 15.50)	4 (0.01 – 10)	5 (0.50 – 12)	5 (0 – 16)	5 (0 – 14)
Mean (SD)	3.31 (2.23)	2.74 (2.10)	4.04 (2.35)	4.71 (2.30)	5.15 (2.26)	5.23 (2.39)
% - Change to previous survey year	-	-17.22	47.45	16.58	9.34	1.55
Screen time – leisure						
Median (Min – Max)	1 (0 – 10)	1 (0 – 8.33)	1 (0 – 4)	2 (0.50 – 10)	2 (0 – 10)	2 (0 – 10)
Mean (SD)	1.79 (1.40)	1.33 (1.13)	1.25 (0.87)	2.25 (1.24)	2.56 (1.34)	2.46 (1.35)
% - Change to previous survey year	-	-25.70	-6.02	80.00	13.78	-3.91
Total screen time						
Median (Min – Max)	4 (0 – 16)	3.5 (0.13 – 15.50)	5 (0.12 – 12)	7 (1.5 – 16)	8 (1.5 – 18)	7 (2 – 16)
Mean (SD)	5.10 (2.61)	4.07 (2.40)	5.29 (2.53)	6.96 (2.50)	7.70 (2.44)	7.69 (2.56)
% - Change to previous survey year	-	-20.20	29.98	31.57	10.63	-0.13

### Trends in screen time: findings from the GLM analysis

All three forms of screen time showed violations of normality (Shapiro-Wilk test, *p* < .01), non-normality of residuals (Q-Q plots) and pronounced positive skewness, as expected for screen time usage [35]. GLMs were applied to analyze the development of screen use across survey years. GLMs with a Gamma distribution and a log link function displayed the best fit across all compared models for all three forms of screen time, reflecting positive skewed data with outliers. The AIC and BIC values of each considered model are shown in Supplement 2. The results of the multicollinearity-analysis are reported in Supplement 3. For GLMs using a log link function eβj​ gives the multiplicative factor, and subtracting 1 then multiplying by 100 expresses it as a percentage relative to the reference group [26]: Percent change = (exp(βj) – 1) x 100%.

A total of *n* = 2538 cases were included in the analysis.

### GLM: screen time – study/work

After adding all coefficients the alternative model demonstrated a significantly better fit than the null model, χ² = 173.855, *p* < .001, with reduced AIC (11057.521) and BIC (11121.751) values compared to the null model (AIC = 11485.839, BIC = 11497.517).

Significant effects were found for the coefficients age (*p* = .010), clinical study phase (*p* < .001), and survey year (2016–2024, all *p*<.05). Medical students in 2016 reported 17.6% less screen time for study- and work reasons compared to those in 2014. In contrast, the survey years 2018, 2020, 2022, and 2024 showed a significant increase in study/work-related screen time compared to 2014 (each *p*<.05), with increases of + 15.0%, + 45.1%, + 56.8%, and + 61.4%, respectively (see Table 3).

**Table 3:** Coefficients of the GLM: screen time study/work

Coefficient	Standard Error	t	95% CI	*p*-value	Effect
Intercept	0.082	10.817	[0.722–1.059]	< 0.001	
Age	0.003	2.589	[0.002–0.016]	0.010	+ 0.9%
Gender (f)	0.025	1.364	[-0.015–0.083]	0.173	+ 3.5%
Living situation (w. others)	0.025	-1.768	[-0.092–0.004]	0.077	-4.2%
Study phase (clinical)	0.026	5.577	[0.086–0.197]	< 0.001	+ 15.7%
Survey year 2016	0.036	-5.406	[-0.264 - -0.124]	< 0.001	-17.6%
Survey year 2018	0.053	2.626	[0.037–0.245]	0.009	+ 15.0%
Survey year 2020	0.039	9.597	[0.296–0.448]	< 0.001	+ 45.1%
Survey year 2022	0.033	13.484	[0.384–0.516]	< 0.001	+ 56.8%
Survey year 2024	0.039	12.136	[0.402–0.557]	< 0.001	+ 61.4%

## GLM: screen time – leisure

After adding the coefficients the alternative model showed a significantly better fit compared to the null model, χ² = 215.509, *p* < .001, and also demonstrated lower AIC (7852.052) and BIC (7916.282) values than the null model (AIC = 8246.171, BIC = 8257.849).

Significant effects were identified for the coefficients age, female gender, living with others, clinical study phase, and survey year (2016–2024), each with *p* < .001.

In 2016 and 2018, screen time for leisure purposes decreased by 24.6% and 23.7%, respectively, compared to 2014 (*p* < .001). In contrast, during the survey years 2020, 2022, and 2024, screen time for leisure purposes increased by 31.1%, 40.4%, and 39.4%, respectively, compared to 2014 (*p*<.001, see Table 4).

**Table 4:** Coefficients of the GLM: screen time leisure

Coefficient	Standard Error	t	95% CI		p-value	Effect
Intercept	0.092	16.182	[1.314 - 1.678]		<.001	
Age	0.004	-8.615	[-0.040- -0.025]		<.001	- 3.2 %
Gender (f)	0.028	-7.605	[-0.269 - -0.159]		<.001	- 19.3%
Living situation (w. others)	0.028	-3.872	[-0.161 - -0.053]		<.001	- 10.1%
Study phase (clinical)	0.029	3.716	[0.051 - 0.167]		<.001	+ 11.5 %
Survey year 2016	0.040	-7.024	[-0.362 - -0.204]		<.001	-24.6 %
Survey year 2018	0.060	-5.036	[-0.419 - -0.183]		<.001	-23.7 %
Survey year 2020	0.043	6.242	[0.186 - 0.357]		<.001	+ 31.1 %
Survey year 2022	0.037	10.116	[0.306 - 0.453]		<.001	+ 40.4 %
Survey year 2024	0.044	7.493	[0.245 - 0.420]		<.001	+39.4 %

### GLM: screen time – total screen time

After adding the coefficients the alternative model provided a significantly better fit than the null model, χ² = 158.810, *p* < .001, and exhibited lower AIC (11836.856) and BIC (11901.087) values compared to the null model (AIC = 12530.703, BIC = 12542.391).

The coefficients for female gender, living with others, clinical study phase, and survey year (except for 2018, *p*=.886) significantly predicted total screen time (each *p*≤.001).

The daily time spent in front of a screen decreased by 20.2% from 2014 to 2016 (*p*<.001).

In contrast, up from 2020 to 2024, the total screen time increased continuously compared to 2014 by + 38.7%, + 51.4%, and + 51.7%, each *p* < .001 (see Table 5).

**Table 5:** Coefficients of the GLM: total screen time

Coefficient	Standard Error	t	95% CI	*p*-value	Effect
Intercept	0.062	27.969	[1.611–1.861]	< 0.001	
Age	0.003	-1.836	[-0.010–4.248 × 10⁻⁴]	0.067	− 0.5%
Gender (f)	0.019	-2.571	[-0.086 - -0.012]	0.010	− 4.8%
Living situation (w. others)	0.019	-3.518	[-0.101 - -0.029]	< 0.001	− 6.3%
Study phase (clinical)	0.020	6.433	[0.088–0.166]	< 0.001	+ 13.5%
Survey year 2016	0.027	-8.359	[-0.279 - -0.173]	< 0.001	− 20.2%
Survey year 2018	0.040	0.144	[-0.072–0.085]	0.886	+ 2.6%
Survey year 2020	0.029	11.212	[0.270–0.385]	< 0.001	+ 38.7%
Survey year 2022	0.025	16.494	[0.366–0.464]	< 0.001	+ 51.4%
Survey year 2024	0.030	14.008	[0.359–0.476]	< 0.001	+ 51.7%

## Discussion

This study investigated how the screen time of medical students at the TUD has developed over the past 10 years. Both reported screen use for study/work reasons and screen time spent for leisure reasons have substantial increased during the observed time span. Similar trends in screen time were observed in other studies among the German population as a whole in the last years [22, 46].

### Screen time study/work

Digitalisation has increased and also found its way into German universities. By 2014, mobile digital devices (such as tablets and laptops), the use of digital media (e.g. electronic articles, PowerPoint presentations) and web-based university organisation platforms were already common. This development has continued in recent years and traditional methods such as paper and pen gradually lost importance in the studies and private. In the last years, digitisation at German universities has been accelerated in particular by the COVID-19 pandemic. Courses had to be converted to digital formats in the short run, which led to an almost complete shift of teaching to the digital space. Even years after the pandemic, digital teaching formats continue to be employed at universities (e.g. lecture videos on online portals instead of face-to-face lectures). This (ongoing utilization) facilitates flexible learning opportunities for both instructors and students, enabling access to educational content at any time and from any location. But, besides the negative effects of spending more screen time, using online platforms is linked to less social interaction and lower attention spans [2] compared to in-person teaching. We observed a decrease in study/work-related screen time from 2014 to 2016. Given the repeated cross-sectional design, this may reflect cohort-specific variation and differences in semester composition between survey waves. In 2016, fewer students from the 6th semester participated compared to 2014 (Supplement 1), which may contribute to lower average study-related screen time.

The largest increase (almost 50%, from 2.74 h to 4.04 h per day) in screen time for study/work among medical students occurred between 2016 and 2018. This effect can be partly explained by methodological factors. In the 2018 survey year, most respondents were in their 10th semester (see Supplement 1) and preparing for their second state examination during the summer term, which likely contributed to an increased amount of screen time for study-related purposes. Despite a balanced semester distribution in the 2020 study cohort, screen time increased again by approximately 17% compared to 2018 (from 4.04 h to 4.71 h). Although participant recruitment was paused during the first lockdown in 2020 to minimize pandemic-related influences [24] it can be assumed that the increased presence of online teaching was already reflected in the data at this point.

By 2024, screen time continued to rise slightly and has since stabilized at around five hours per day spent on study- and work-related screen activities. While medical students increasingly use digital learning methods and engage in digital environments such as lectures and clinical simulations, several reviews suggest, that prolonged screen time—even for educational purposes—can have negative effects on academic outcomes. For instance Amez and Baert [3] found, that vast majority of studies reported a significantly negative association between smartphone use, including for study purposes and academic performance. This is supported by a more recent meta-analysis, which also concluded, that there is a statistically significant negative correlation between problematic smartphone use and academic achievement [33]. These findings suggest that elevated screen time intended for learning can become counterproductive thus, when promoting compulsive behaviors or cognitive/emotional fatigue (also known as digital fatigue) [3, 33].

### Screen time leisure

The results indicate that leisure-related screen time initially declined between 2014 and 2018, before increasing substantially thereafter. This initial decrease may reflect a phase of digital saturation. Technological innovations often lead to a temporary peak in usage, followed by a period of normalization or more mindful media consumption. Following the initial appeal of newly available mobile devices, it is conceivable that the novelty effect had largely diminished by 2018. The decline could also have methodological reasons, as only the 10th semester was surveyed in 2018.

Between 2018 and 2020, an 80% increase in leisure-related screen time was observed (from 1.25 h to 2.25 h). This period coincides—analogous to the trend in study/work-related screen time—with the onset of the COVID-19 pandemic, which has been associated with changes in leisure behavior and increased media consumption in the general population [43]. In addition, this increase could be explained by the growing prevalence of digital entertainment and communication platforms. For example, the platform TikTok, which is particularly popular among young people has over 20 million active monthly users [5], was launched in Germany in 2018.

Between 2020 and 2024, leisure-related screen time continued to rise and has stabilized at approximately 2.5 h per day since 2022. During this period, additional short video platforms such as Instagram Reels (2020) and YouTube Shorts (2021) became established alongside TikTok. Recent studies indicate that short video formats possess a particularly addictive entertainment character [34]. The associated user behavior is increasingly discussed in the literature under the term Short-Video Addiction (SVA) [29, 34]. A central feature of these platforms is the use of so-called engagement-optimized algorithms, which aim to keep users on the respective platforms for as long as possible [28, 50]. The widespread availability of smartphones and the steadily increasing volume of mobile data have made the use of such services possible virtually anytime and anywhere. This increase in leisure-related screen use observed among medical students reflects a broader trend in the general population in Germany, where screen-based leisure activities have risen substantially between 2014 and 2024 (Freizeitmonitor, [16]).

The results of the GLMs underscore the significance of the trend identified in the descriptive analysis. The explanatory contribution of the survey years—particularly between 2020 and 2024—compared to the baseline year 2014 clearly exceeds that of sociodemographic factors. This effect is more pronounced for screen time related to study and work purposes than for leisure-related screen time.

### Implications & solutions

The observed increase in screen time warrants critical consideration in light of its potential health implications. Extensive screen exposure is associated with a range of physiological and psychological effects, which vary depending on individual disposition [38], usage context [35] and additional contextual factors such as duration or timing [18]. Screen time is closely linked to a sedentary lifestyle, which is an established risk factor for cardiovascular disease, obesity, and metabolic syndrome [49]. According to a meta-analysis by [20] five to six hours of daily screen use carries a cardiovascular risk similar to that of prolonged sitting for ten to eleven hours without interruption. Further meta-analyses and systematic reviews have identified associations between increased screen use and digital eye strain [21] or the development of myopia [14]. Similarly, increased screen use is associated with reduced sleep quality [17]. At the mental health level, numerous studies have reported associations between excessive screen time and increased risk for depression, anxiety disorders, and reduced psychological well-being [36]. As for sleep health, rather than screen time per se, specific usage characteristics (e.g., timing, content, emotional involvement [9, 12, 19], in interaction with individual vulnerabilities (e.g., coping style, attentional biases, executive functioning), as described in the I-PACE model [6] are decisive for how screen use interacts with sleep behaviour, while relationships can often be bidirectional [44]. The observed increase primarily reflects study/work-related use, which likely indicates structural digitalization of education; thus, health risks may depend less on total screen hours per se than on usage patterns such as prolonged uninterrupted exposure, evening use, and concurrent sedentary behavior.

Effective risk reduction requires the combination of behavioural and structural prevention strategies. University should increase the awareness of the ubiquity of screen use and its potential adverse health effects - among both staff and students. Greater awareness in medical students is doubly valuable: it can enhance their own well-being and, in turn, improve population health, because physicians’ personal habits shape the advice they give and the credibility of their role-model function [7, 30].

As digital methods are increasingly integrated into educational settings, the resulting rise in educational screen time calls for evidence-based countermeasures. One practical approach to mitigate negative effects on eye health is incorporating the 20-20-20 rule into lectures and self-study sessions: every 20 min, look at something 20 feet away for 20 s [40]. Additionally, there is an ongoing debate in the literature about whether handwritten or digital note-taking leads to better academic performance. The most recent meta-analysis [13] found a small but statistically significant advantage for handwriting, although contextual factors—especially digital distractions such as email or social media—appear to play a crucial role [48]. Therefore, online learning platforms should offer focus modes to support distraction-free digital learning.

Furthermore, comprehensive media literacy should include awareness of personal health behaviors related to screen use. Evidence-based guidelines can help define limits and preventive strategies, while occupational health policies should regulate screen time through defined maximum durations, mandatory breaks, and ergonomic standards to reduce long-term strain. The implications and recommendations formulated can be discussed with the student council of TU Dresden and the patient advisory board of the Department of General Medicine in order to achieve implementation of the results.

Lastly, it is worth to mentioned that the impact of the COVID-19 pandemic on the health behavior of the sample presented here was analyzed in the publication by Liebig et al., [25]. The longitudinal analyses showed an increase in physical activity and a reduction in the consumption of unhealthy foods. Screen time, sleep quality, and substance use (alcohol and tobacco) remained unchanged [25]. However, the extent to which the COVID-19 pandemic affected screen time use should be investigated in further studies.

### Limitations

The study is subject to several limitations. The surveyed populations partly differed in their semester distribution across the respective years, which likely influenced screen use levels. It cannot be ruled out that the switch from pen and paper to an online survey may have influenced the response rate.

Moreover, the study represents a series of repeated cross-sectional analyses rather than a true longitudinal design with within-person comparisons. Based on earlier work [25] approx. 10% of respondents are expected to participate in two consecutive survey waves. Such within-panel overlap can deflate standard errors because observations are no longer fully independent. Assuming a plausible intra-individual correlation of ρ = 0.4–0.6, the resulting design effect inflates the standard errors by about 3% to 6%. Consequently, any bias resulting from the overlap is modest and does not alter the substantive conclusions.

Recall bias of daily spent screen time might differ between survey years as modern devices such as Iphones give exact feedback on daily spent screen time. Furthermore, since the covid-19 pandemic, screen time as a parameter of health behaviour became more important and more aware in peoples mind than it was before.

In addition, the MSHS items used between 2014 and 2024 did not capture detailed usage contexts - such as a fully differentiated assessment of leisure activities - nor did they provide an activity-level breakdown within the study/work domain (e.g., specific learning-related digital activities), and they did not account for mixed or simultaneous use (e.g., studying while streaming entertainment). Consequently, we cannot retrospectively determine which study-related digital activities contributed to the observed trends or their role in learning. Furthermore, a 2024 think-aloud pre test of more detailed items highlighted challenges in defining valid categories; therefore, these items were not implemented.

Finally, no health-related variables were collected continuously and in a harmonized manner over the ten years, which is why it was not possible to calculate any associations with, for example, mental health. At the same time, we were already able to show associations with screen time and sleep quality in other publications on the cohort [24]. Our primary goal was to show a 10-year trend.

## Conclusions

Screen time among medical students has substantial increased over the survey period, with both study/work-related and leisure-related use contributing substantially to this increase. Negative effects of screen use are diverse, often bidirectional and depend on individual and contextual factors. Prevention strategies and public awareness are needed to mitigate potential health consequences. Future studies should continue to monitor these trends and ideally examine usage patterns alongside health outcomes. It is also interesting to examine the interplay between screen time, mental health, and academic performance in more detail. Furthermore, survey measures with finer-grained approaches (e.g., EMA and/or objective device/app logs) should be used to capture activity types an simultaneity.

## Supplementary Information

Supplementary Material 1.

## Data Availability

The raw data supporting the conclusions of this article will be made available by the authors, without undue reservation. To ensure compliance with the data protection plan outlined in the ethics application, all individuals requiring access to the data must first be reported to the Ethics Committee of TU Dresden (No. EK 15012014). Upon notification and signing a data protection agreement, access to the data will be granted based on justified requests.
