# Supplementary material for: Ten years of screen time among medical students in Dresden, Germany: unveiling the trends
Source: BMC Med Educ. 2026 Mar 30;26:581. doi: 10.1186/s12909-026-09024-x (PMC13063611; doi:10.1186/s12909-026-09024-x)
Supplement: Supplementary file 1 — Supplementary Material 1. [file 12909_2026_9024_MOESM1_ESM.docx]

# Supplement 1 - Response rate and distribution of semesters

|  | 2014 | 2016 | 2018 | 2020 | 2022 | 2024 |
| --- | --- | --- | --- | --- | --- | --- |
| Number of matriculated students (2^nd^, 6^th^, 10^th^ semester), n | 697 | 706 | - | - | - | - |
| Number of matriculated students  (10^th^ semester), n | - | - | 320 | - | - | - |
| Number of matriculated students (all semesters), n | - | - | - | 1418 | 1986 | 1997 |
| Number of received questionnaires, n | 607 | 497 | 177 | 476 | 720 | 507 |
| **Response Rate** | **87.1%** | **70.4%** | **55.3%** | **33.6%** | **36.3%** | **25.4%** |
| Semester, n |  |  |  |  |  |  |
| 1 | 2 | 1 | - | 21 | 3 | 2 |
| 2 | 209 | 191 | - | 50 | 172 | 101 |
| 3 | - | - | - | 32 | 8 | - |
| 4 | 6 | 1 | - | 39 | 51 | 38 |
| 5 | 1 | - | - | 36 | 3 | 2 |
| 6 | 159 | 86 | - | 46 | 157 | 91 |
| 7 | 2 | - | - | 25 | 13 | 4 |
| 8 | 1 | 2 | - | 56 | 124 | 37 |
| 9 | 6 | 2 | 1 | 19 | 3 | 1 |
| 10 | 191 | 189 | 150 | 49 | 94 | 69 |
| >10 | 6 | 6 | 7 | 39 | 23 | 8 |
| Missings, n | 2 | - | - | 4 | 4 | 11 |
| **Total, n** | **585** | **478** | **158** | **416** | **655** | **364** |

# Supplement 2 - AIC and BIC values for all models considered

**Screen Time Work**

Gamma Log X

| *Model Summary - ScreenTimeWorkGLM* | | | | | | | | | | | | | |
| --- | --- | --- | --- | --- | --- | --- | --- | --- | --- | --- | --- | --- | --- |
| Model | | Deviance | | AIC | | BIC | | df | | Χ² | | p | |
| H₀ |  | 1145.394 |  | 11485.839 |  | 11497.517 |  | 2537 |  |  |  |  |  |
| H₁ |  | 971.539 |  | 11057.521 |  | 11121.751 |  | 2528 |  | 173.855 |  | < .001 |  |
|  | | | | | | | | | | | | | |

Gamma Inverse

| *Model Summary - ScreenTimeWorkGLM* | | | | | | | | | | | | | |
| --- | --- | --- | --- | --- | --- | --- | --- | --- | --- | --- | --- | --- | --- |
| Model | | Deviance | | AIC | | BIC | | df | | Χ² | | p | |
| H₀ |  | 1145.394 |  | 11485.839 |  | 11497.517 |  | 2537 |  |  |  |  |  |
| H₁ |  | 984.164 |  | 11092.363 |  | 11156.594 |  | 2528 |  | 161.230 |  | < .001 |  |
|  | | | | | | | | | | | | | |

Gaussian

| *Model Summary - ScreenTimeWorkGLM* | | | | | | | | | | | | | |
| --- | --- | --- | --- | --- | --- | --- | --- | --- | --- | --- | --- | --- | --- |
| Model | | Deviance | | AIC | | BIC | | df | | Χ² | | p | |
| H₀ |  | 15358.007 |  | 11775.593 |  | 11787.272 |  | 2537 |  |  |  |  |  |
| H₁ |  | 12771.197 |  | 11325.473 |  | 11389.704 |  | 2528 |  | 2586.810 |  | < .001 |  |
|  | | | | | | | | | | | | | |

Gaussian Inverse

| *Model Summary - ScreenTimeWorkGLM* | | | | | | | | | | | | | |
| --- | --- | --- | --- | --- | --- | --- | --- | --- | --- | --- | --- | --- | --- |
| Model | | Deviance | | AIC | | BIC | | df | | Χ² | | p | |
| H₀ |  | 1224.516 |  | 14520.171 |  | 14531.849 |  | 2537 |  |  |  |  |  |
| H₁ |  | 1183.483 |  | 14451.665 |  | 14515.895 |  | 2528 |  | 41.033 |  | < .001 |  |
|  | | | | | | | | | | | | | |

**Screen Time Leisure**

Gamma Log X

| *Model Summary - ScreenTimeLeisureGLM* | | | | | | | | | | | | | |
| --- | --- | --- | --- | --- | --- | --- | --- | --- | --- | --- | --- | --- | --- |
| Model | | Deviance | | AIC | | BIC | | df | | Χ² | | p | |
| H₀ |  | 1560.343 |  | 8246.171 |  | 8257.849 |  | 2537 |  |  |  |  |  |
| H₁ |  | 1344.834 |  | 7852.052 |  | 7916.282 |  | 2528 |  | 215.509 |  | < .001 |  |
|  | | | | | | | | | | | | | |

Gamma Inverse

| *Model Summary - ScreenTimeLeisureGLM* | | | | | | | | | | | | | |
| --- | --- | --- | --- | --- | --- | --- | --- | --- | --- | --- | --- | --- | --- |
| Model | | Deviance | | AIC | | BIC | | df | | Χ² | | p | |
| H₀ |  | 1560.343 |  | 8246.171 |  | 8257.849 |  | 2537 |  |  |  |  |  |
| H₁ |  | 1353.788 |  | 7870.349 |  | 7934.580 |  | 2528 |  | 206.555 |  | < .001 |  |
|  | | | | | | | | | | | | | |

Gaussian

| *Model Summary - ScreenTimeLeisureGLM* | | | | | | | | | | | | | |
| --- | --- | --- | --- | --- | --- | --- | --- | --- | --- | --- | --- | --- | --- |
| Model | | Deviance | | AIC | | BIC | | df | | Χ² | | p | |
| H₀ |  | 4706.657 |  | 8774.004 |  | 8785.683 |  | 2537 |  |  |  |  |  |
| H₁ |  | 3909.495 |  | 8321.029 |  | 8385.259 |  | 2528 |  | 797.162 |  | < .001 |  |
|  | | | | | | | | | | | | | |

Gaussian Inverse

| *Model Summary - ScreenTimeLeisureGLM* | | | | | | | | | | | | | |
| --- | --- | --- | --- | --- | --- | --- | --- | --- | --- | --- | --- | --- | --- |
| Model | | Deviance | | AIC | | BIC | | df | | Χ² | | p | |
| H₀ |  | 4706.657 |  | 8774.004 |  | 8785.683 |  | 2537 |  |  |  |  |  |
| H₁ |  | 3933.105 |  | 8336.310 |  | 8400.541 |  | 2528 |  | 773.551 |  | < .001 |  |
|  | | | | | | | | | | | | | |

**Screen Time Total**

Gamma Log X

| *Model Summary - TST_GLM* | | | | | | | | | | | | | |
| --- | --- | --- | --- | --- | --- | --- | --- | --- | --- | --- | --- | --- | --- |
| Model | | Deviance | | AIC | | BIC | | df | | Χ² | | p | |
| H₀ |  | 670.936 |  | 12530.703 |  | 12542.381 |  | 2537 |  |  |  |  |  |
| H₁ |  | 512.126 |  | 11836.856 |  | 11901.087 |  | 2528 |  | 158.810 |  | < .001 |  |
|  | | | | | | | | | | | | | |

Gamma Inverse

| *Model Summary - TST_GLM* | | | | | | | | | | | | | |
| --- | --- | --- | --- | --- | --- | --- | --- | --- | --- | --- | --- | --- | --- |
| Model | | Deviance | | AIC | | BIC | | df | | Χ² | | p | |
| H₀ |  | 670.936 |  | 12530.703 |  | 12542.381 |  | 2537 |  |  |  |  |  |
| H₁ |  | 517.495 |  | 11864.215 |  | 11928.446 |  | 2528 |  | 153.441 |  | < .001 |  |
|  | | | | | | | | | | | | | |

Gaussian

| *Model Summary - TST_GLM* | | | | | | | | | | | | | |
| --- | --- | --- | --- | --- | --- | --- | --- | --- | --- | --- | --- | --- | --- |
| Model | | Deviance | | AIC | | BIC | | df | | Χ² | | p | |
| H₀ |  | 20992.851 |  | 12568.833 |  | 12580.511 |  | 2537 |  |  |  |  |  |
| H₁ |  | 15516.869 |  | 11819.711 |  | 11883.942 |  | 2528 |  | 5475.982 |  | < .001 |  |
|  | | | | | | | | | | | | | |

Gaussian Inverse

| *Model Summary - TST_GLM* | | | | | | | | | | | | | |
| --- | --- | --- | --- | --- | --- | --- | --- | --- | --- | --- | --- | --- | --- |
| Model | | Deviance | | AIC | | BIC | | df | | Χ² | | p | |
| H₀ |  | 20992.851 |  | 12568.833 |  | 12580.511 |  | 2537 |  |  |  |  |  |
| H₁ |  | 15595.960 |  | 11832.615 |  | 11896.845 |  | 2528 |  | 5396.891 |  | < .001 |  |
|  | | | | | | | | | | | | | |

# Supplement 3 - Multicollinearity analysis

|  | | | | | |
| --- | --- | --- | --- | --- | --- |
|  | | Tolerance | | VIF | |
| Survey year |  | 0.942 |  | 1.062 |  |
| Study phase |  | 0.833 |  | 1.200 |  |
| Gender |  | 0.980 |  | 1.021 |  |
| Living situation |  | 0.980 |  | 1.021 |  |
| Age |  | 0.831 |  | 1.203 |  |
